# Eccrine poroma versus eccrine porocarcinoma: a comparative study of clinicopathological features^☆^

**DOI:** 10.1016/j.abd.2024.04.010

**Published:** 2024-12-12

**Authors:** Qin-Xiao Wang, Si-Yu Luo, Kai-Yi Zhou, Xiao Shen, Sheng Fang

**Affiliations:** a Department of Dermatology, the First Affiliated Hospital of Chongqing Medical University, Chongqing, China; b Department of Plastic and Burn Surgery, The First Affiliated Hospital of Chongqing Medical University, Chongqing, China

**Keywords:** Clinical conference, Porocarcinoma, Pathology, clinical, Poroma

## Abstract

**Background:**

Eccrine porocarcinoma (EPC) is a rare cutaneous neoplasm, commonly arising from its benign counterpart, eccrine poroma (EP), but potential unrevealed clinicopathological differences between them are not well understood.

**Objectives:**

This study aimed to identify clinicopathological features of EP and EPC and describe the factors that may be associated with the malignant transformation of EP by comparing the two groups.

**Methods:**

A total of 37 cases of EP and 22 cases of EPC diagnosed between January 2017 and June 2023 were retrospectively reviewed, and the clinical and histopathological characteristics were compared using statistical methods.

**Results:**

Clinical and histopathologic data such as age, gender, site, clinical presentation, and histopathologic characteristics were collected. The EPC group was more common in older patients, with more cases located in exposed areas, and the patients with EPC had larger lesions with a higher incidence of ulceration. Histopathological features showed significant differences in tumor architecture, ulceration, squamous differentiation, spindle cell changes, central necrosis, and diffuse inflammatory infiltration between the two groups.

**Study limitations:**

This study has limitations due to a small number of cases with potential experimental bias.

**Conclusion:**

The clinicopathological features of EP and EPC were compared in this study and the results may assist clinicians in diagnosis and management of these tumors by helping to identify potential factors associated with the malignant transformation of EP.

## Introduction

Eccrine Porocarcinoma (EPC), also known as malignant eccrine poroma, is a rare malignant adnexal tumor of the skin originating from the eccrine gland. This neoplasm was first recognized by Pinkus & Mehregan in 1963 as “epidermotropic eccrine carcinoma”.^1^ It accounts for 0.005% to 0.01% of all malignant cutaneous neoplasms.^2^, ^3^ Although this neoplasm is rare, it is still the most frequently malignant tumor of eccrine gland.^4^ Studies have shown that some EPCs are developed from their preexisting benign counterpart, Eccrine Poroma (EP).^5^, ^6^, ^7^ The exact etiology and pathogenesis of EPC are still unknown, and the factors that are associated with the malignant transformation of EPC have not yet been identified.^8^, ^9^ Furthermore, EPC is a rare eccrine sweat gland malignancy that is often clinically misdiagnosed, and the differential diagnosis of EPC is extensive pathologically, even sometimes the differences between EP and EPC are not straightforward. To shed light on this, the authors present a series of cases and highlight the key features of the two rare diseases. Apart from cytological malignant features, the authors focus on subtle, less commonly encountered pathological features of EPC versus EP.

## Materials and methods

### Materials

The authors undertook a retrospective review of 37 cases of EP and 22 cases of EPC that had been clinicopathologically diagnosed between January 2017 and June 2023 in the Department of Dermatology, the First Affiliated Hospital of Chongqing Medical University. The diagnosis of EP was based on histopathology characterized by tumor clusters composed of poroma cells connected to the epidermis with ductal lumina. Poroma cells are monomorphic, small, cuboidal with basophilic round nuclei, inconspicuous nucleoli, and compact eosinophilic cytoplasm. The diagnosis of EPC was based on diffuse malignant clusters of tumor cells with nuclear and cytoplasmic pleomorphism, nuclear hyperchromatism, and mitotic activity. The sections stained with Hematoxylin and Eosin (H&E) were obtained from the Department of Dermatology, the First Affiliated Hospital of Chongqing Medical University. Clinical data were gathered from inpatient medical records and pathology request forms. Ethics approval was granted by the Ethics Committee of the First Affiliated Hospital of Chongqing Medical University (K2023-351), with written consent waived because only de-identified information was used for analysis.

### Methods

The pathology archives of skin biopsies containing the terms “eccrine poroma” and “eccrine porocarcinoma” in the final diagnosis were searched. Biopsies that met the inclusion criteria were reviewed independently by three experienced dermatopathologists. The following histopathological features were examined for the eccrine poroma and eccrine porocarcinoma, including types of growth pattern (infiltrating, in situ); tumor architecture (symmetry, asymmetry); ulceration; sharp demarcation between normal keratinocytes and poroma cells; squamous differentiation; spindle cell change; central necrosis; degree of inflammatory infiltration (diffuse, focal); delicate fibrovascular stroma, decapitation secretion and sebaceous differentiation. Clinical data including gender, age, anatomical distribution, size, and the presence of ulceration were analyzed. A comparison was made of the clinicopathological characteristics of the two groups.

### Statistical analyses

All the descriptive data including the clinical and histopathological data were generated for all variables. Data management and statistical analyses were carried out by using SPSS software (version 23.0). Age was expressed as the mean (range) age and was compared using Student’s *t*-test. The χ²-test and Fisher’s exact test were conducted to assess the association between the categorical variables, such as sex, or location of the tumor; p-values less than 0.05 were considered statistically significant.

## Results

### Demographic and clinical characteristics

**Figure 1:**
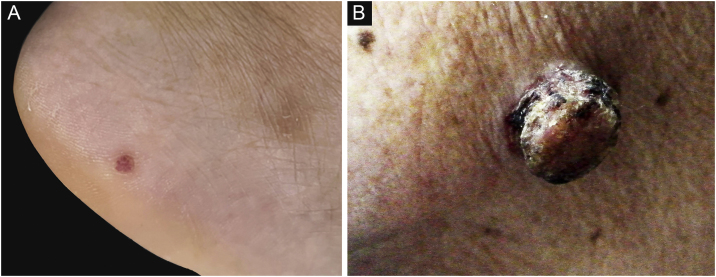
Clinical image of EP and EPC. (A) EP manifested as a well-circumscribed, small papular on the foot. (B) EPC manifested as a dark red nodule on the face with ulceration and crusting.

The mean age at diagnosis was 53.0 years for EP and 69.5 years for EPC. The age of the EPs ranged from 16 to 91 years and the age of the EPCs ranged from 50 to 93 years. There was a significant difference between the age groups (p = 0.001). Of the 37 patients in the EP group, 13 (13/37, 35.1%) were male and 24 (24/37, 64.9%) female; of the 22 patients in the EPC group, 10 (10/22, 45.5%) were male and 12 (12/22, 54.5%) female. There was no significant gender difference (p > 0.05) . The clinical data of the patients are presented in Table 1. 45.5% of EPCs were located in the exposure area, 10 cases on the head and neck, while in the EP group, only 10.8% were located in the exposure area, 2 on the scalp and 2 on the face (Fig. 1). In the EPC group, 14 patients (14/22, 63.6%) had ulceration, whereas only 2 (2/22, 5.4%) were in the EP group. Lesion size was categorized as <1 cm, 1‒1.5 cm, >1.5 cm, with EP patients accounting for 64.9%, 29.7% and 5.4% and EPC patients accounting for 9.1%, 36.4% and 54.5% respectively. Significant differences were found between the two groups for lesion location, presence of ulceration, and lesion size.

**Table 1:** Demographic characteristics and Clinical characteristics of Patients with EP and EPC.

		EP, n (%)	EPC, n (%)	p-value
**Age**	Range	16‒91	50‒93	0.001
	Mean	53.0	69.5	
**Gender**	Female	24 (64.9%)	12 (54.5%)	0.432
	Male	13 (35.1%)	10 (45.5%)	
**Location**	Exposure	4 (10.8%)	10 (45.5%)	0.001
	Head	2	9	
	Neck	2	1	
	Non-exposure	33(89.2%)	12 (54.5%)	
	Trunk	3	2	
	Extremities	30	10	
**Ulceration**	Yes	2 (5.4%)	14 (63.6%)	0.000
	No	35 (94.6%)	8 (36.4%)	
**Size**	<1 cm	24 (64.9%)	2 (9.1%)	0.000
	1‒1.5 cm	11 (29.7%)	8 (36.4%)	
	>1.5 cm	2 (5.4%)	12 (54.5%)	

EP, Eccrine Poroma; EPC, Eccrine Porocarcinoma.

### Histopathologic findings

**Figure 2:**
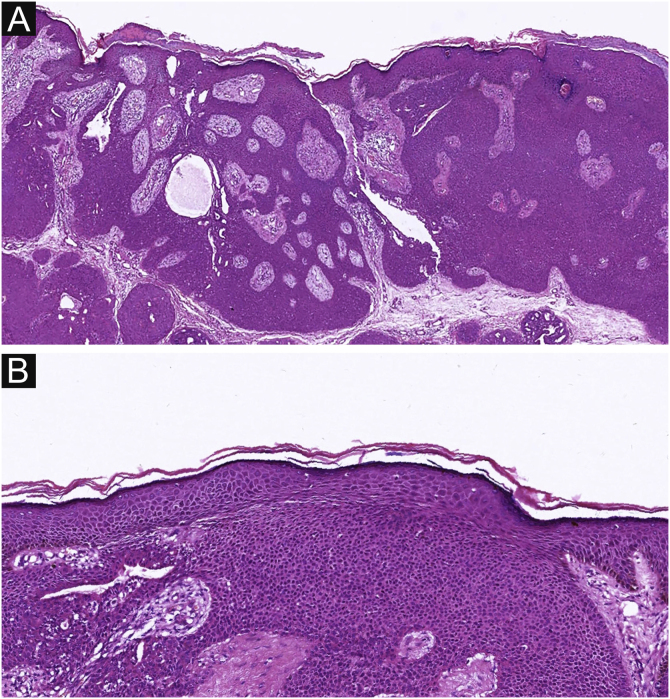
The histological appearance of EP. (A) well-defined neoplasm connected to the epidermis. (B) The oncocytes are cytologically bland without ulceration. A, 20×; B, 100×.

**Figure 3:**
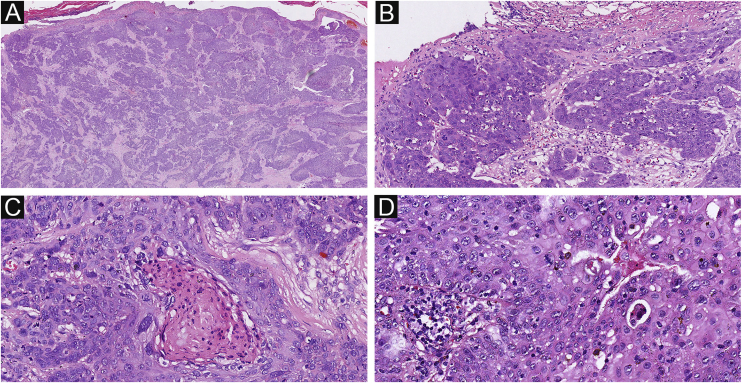
The histological appearance of EPC. (A) A neoplasm with infiltrating growth pattern. (B) The malignant cells have hyperchromatic, disorderly arrangement, and atypical nuclei with mitoses,ductal structures and ulceration. (C) Some show area of central necrosis and cytological atypia. (D) Tumor necrosis, disorderly arrangement and mitoses are present. Original magnifications: A, 20×; B, 100×; C, 200×; D, 200×.

**Figure 4:**
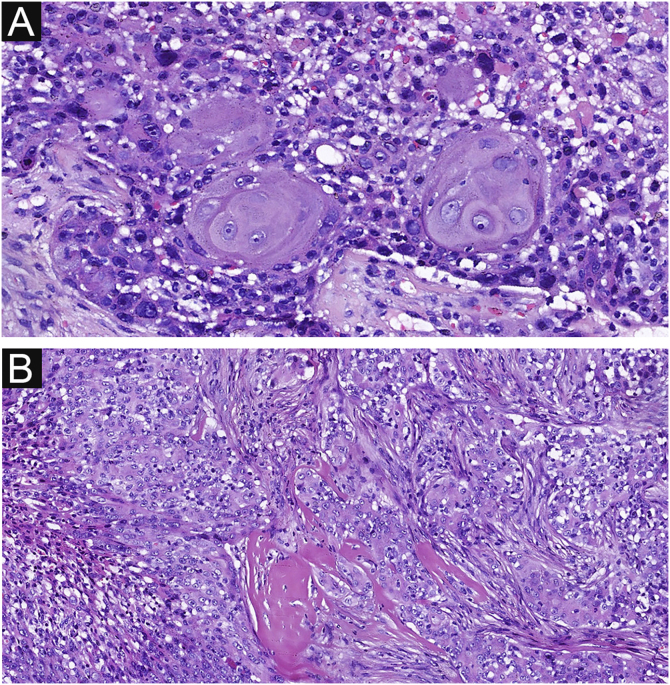
Focal squamous differentiation and spindle changes in EPC. (A) Squamous differentiation. (B) Spindle changes. Original magnifications: A, 200×; B, 100×.

Of the 37 patients with EP, none showed an infiltrative growth pattern, while 30 patients (30/37, 81.1%) had a symmetrical tumor architecture (Fig. 2). In contrast, 20 of the EPC patients showed an invasive growth pattern, the other two were carcinoma in situ, and 16 patients (16/22, 72.7%) had asymmetric tumor structures. (Fig. 3A). Among the patients with EP, 3 patients (3/37, 8.1%) presented with ulceration, and there were 20 patients (20/22, 90.9%) with ulceration in the EPC group (Fig. 3B). “Central necrosis” refers to the central necrotic or dead material within the tumor. Four patients (4/37, 10.8%) with an EP and twelve patients (12/22, 54.5%) with an EPC had this feature (Fig. 3C). In the present study, the tumor cells in the EPC group had squamous differentiation and spindle changes in 8 and 4 cases respectively (Fig. 4), whereas these changes were less frequent in the EP group, 2 and 1 cases respectively. In addition, diffuse inflammatory infiltrates were significantly more prevalent in the EPC group, with eight patients (8/22, 36.4%), compared to only one (1/37, 2.7%) in the EP tissue. In addition, well-established histopathological differences between the two groups were significantly different in this study. These included cell arrangement, cell mitoses, and cytological atypia.

**Figure 5:**
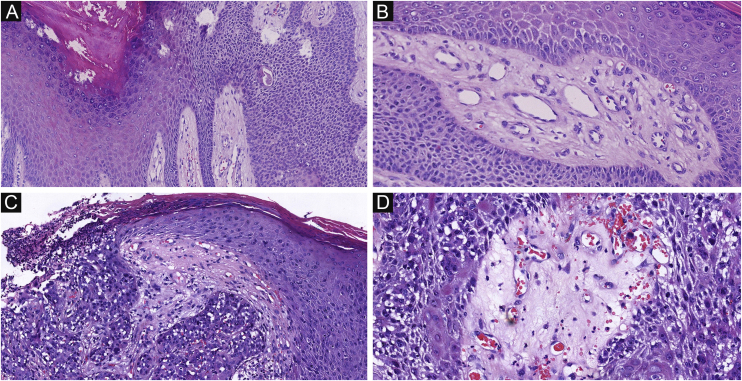
Delicate fibrovascular stroma and sharp demarcation of EP and EPC. (A) Normal keratinocytes and well-formed poroma cells are seen in this field, with a clear demarcation between them in EP. (B) A delicate fibrovascular stroma of EP. (C) This lesion shows a sharp demarcation between normal keratinocytes and poroma cells with nuclear pleomorphism and hyperchromatism in EPC. (D) A delicate fibrovascular stroma of EPC. Original magnifications: A, 100×; B, 200×; C, 100×; D, 200×.

Delicate fibrovascular stroma and sharp demarcation between normal keratinocytes and poroma cells are important pathological features of poroma, seen in 37 and 33 cases respectively in this study (Fig. 5 A‒B). This feature was also common in EPCs, seen in 20 and 18 cases respectively (Fig. 5 C‒D), with no significant difference between the two groups. Decapitation secretion and sebaceous differentiation are infrequent features of EP. No significant difference was found between the two groups in terms of sebaceous differentiation and decapitation secretion in the present study. There are two types of poroma and porocarcinoma: eccrine and apocrine. Only apocrine poromas and porocarcinomas have sebaceous and decapitation secretions (apocrine type) because these glands are related to the pilo-sebaceous-apocrine unit. The location of the tumors on apocrine glandular areas could be an indication of apocrine differentiation. Histopathological findings are shown in Table 2.

**Table 2:** Histopathologic features of patients with EP and EPC.

		EP, n (%)	EPC, n (%)	p-value
**Growth pattern**	Infiltrating	0 (0.0%)	20 (90.9%)	0.000
	In situ	37 (100.0%)	2 (9.1%)	
**Tumor architecture**	Symmetry	30 (81.1%)	6 (27.3%)	0.000
	Asymmetry	7 (18.9%)	16 (72.7%)	
**Ulceration**	Yes	3 (8.1%)	20 (90.9%)	0.000
	No	34 (91.9%)	2 (9.1%)	
**Sharp demarcation**	Yes	33 (89.2%)	18 (81.8%)	0.424
	No	4 (10.8%)	4 (18.2%)	
**Mature duct formation**	Yes	35 (94.6%)	22 (100.0%)	0.267
	No	2 (5.4%)	0 (0.0%)	
**Squamous differentiation**	Yes	2 (5.4%)	8 (36.4%)	0.002
	No	35 (94.6%)	14 (63.6%)	
**Spindle cell change**	Yes	1 (2.7%)	4 (18.2%)	0.039
	No	36 (97.3%)	18 (81.8%)	
**Cell arrangement**	Disordered	4 (10.8%)	22 (100.0%)	0.000
	Ordered	33 (89.2%)	0 (0.0%)	
**Central necrosis**	Yes	4 (10.8%)	12 (54.5%)	0.000
	No	33 (89.2%)	10 (45.5%)	
**Cell Mitoses**	Yes	1 (2.7%)	22 (100.0%)	0.000
	No	36 (97.3%)	0 (0.0%)	
**Cytological atypia**	Yes	4 (10.8%)	22 (100.0%)	0.000
	No	33 (89.2%)	0 (0.0%)	
**Diffuse inflammatory infiltration**	Yes	1 (2.7%)	8 (36.4%)	0.001
	No	36 (97.3%)	14 (63.6%)	
**Delicate fibrovascular stroma**	Yes	37 (100.0%)	20 (90.9%)	0.062
	No	0 (0.0%)	2 (9.1%)	
**Decapitation secretion**	Yes	2 (5.4%)	4 (18.2%)	0.116
	No	35 (94.6%)	18 (81.8%)	
**Sebaceous differentiation**	Yes	1 (2.7%)	2 (9.1%)	0.280
	No	36 (97.3%)	20 (90.9%)	

EP, Eccrine Poroma; EPC, Eccrine porocarcinoma.

## Discussion

EPC is a relatively rare malignant cutaneous neoplasm arising from the sweat glands. As a malignant counterpart of EP, its incidence rate was about 1.866 per 100,000 according to the previous study.^10^ In recent years, the incidence rate of EPC has been reported to be gradually increasing. In the present study, EPC accounted for about 0.008% of all skin tumors, the incidence may be influenced by consulting cases which may increase the incidence. Local recurrences and lymph node metastasis are seen in about 17% of cases, and in 8% of patients may lead to death due to disseminated malignancy.^10^ According to previous studies, although EPC can be primary, most of them are derived from EP, and the associated factors for the development of EP into EPC are still unclear. The proportion of EPC cases developing from EP has varied in previous reports. In this case series, 4 patients (4/22, 18.2%) presented with sudden onset of ulceration, hemorrhage, or enlargement at the sites of pre-existing lesions, whereas 6 patients (6/22, 27.3%) had residual foci of benign eccrine lesions. These findings suggest that these EPC cases may have originated from pre-existing EP. However, some cases may have developed over a longer time, making it difficult to distinguish the transitional elements. In addition, 4 cases (4/22, 18.2%) of EPC patients in the present study developed metastases, in agreement with previous studies.

Previous studies found that chronic light exposure is a risk factor for developing EPC.^5^, ^6^, ^7^ This may be related to its effect on the regulation of the immune system. UVB induces and activates immunosuppressive regulatory T-cells, reduces the number and function of Langerhans cells, and increases the release of immunosuppressive mediators.^8^ Recent reports have debated the common location of EPCs. Some studies have shown that the most common location of EPC is the lower limb, but others have suggested that the most common location is the head neck, or trunk.^5^, ^11^, ^12^, ^13^ These results showed that EPCs were predominantly located in the exposure area. This finding supports that chronic light exposure is one of the possible associated factors for malignant transformation. The difference in the mean age between the two groups suggests that elderly people are more likely to have a malignant transformation which may be due to reduced immune surveillance. While some previous case reports suggest that EPCs may be associated with larger size, actinic damage and older age, the present study provides additional evidence.

Statistically significant differences were observed in terms of disordered cell arrangement, cellular mitosis, and cytological atypia according to the diagnostic and inclusion criteria for EPC in this study. Apart from cytological malignant features, the authors focus on less commonly encountered pathological features of EPC versus EP. Infiltrative growth patterns and asymmetric tumor architecture were significantly more common in the EPC group, similar to the differences between most benign and malignant tumors. Some researchers believe that the aggressive biological behavior associated with ulceration may be the consequence of an intrinsic biological property of the tumor, which favors its dissemination and is locally manifested by the absence of epidermal integrity.^14^ The data supported this finding that ulceration was more common in the EPC group. Similar findings have been reported in cutaneous squamous cell carcinoma and melanoma.^14^, ^15^, ^16^, ^17^ Central necrosis refers to the death of central cancer cells with surrounding inflammatory debris and the surrounding inflammation results in a comedo-like appearance.^5^ The authors observed a central necrosis pattern in 54.5% of the EPC cases, which is higher than the 32% and 45% observed by the previous researchers.^5^, ^12^ This aspect may be due to localized ischemia and necrosis in the malignant tumor, leading to cell death and aggregation in the central area, forming a central necrotic zone. In the EPC group, the authors observed a significantly higher incidence of diffuse inflammatory infiltration of the dermis than in the EP group. Tumor-associated inflammation is a feature of essentially all cancers and can confer tumor-promoting function.^18^, ^19^ This may be due to the highly active and invasive nature of the tumor, leading to an increased inflammatory response in the surrounding tissue.

In the EPC group, the authors identified squamous differentiation in 36.4% of patients. This is similar to the findings of Riera-Leal et al.^12^ The presence of squamous differentiation in EPC has been a subject of varied interpretations. Some scholars considered it a distinct pathological subtype of EPC, while others viewed it as a variant of squamous cell carcinoma.^12^, ^20^ There was also a perspective that deemed it as an incidental finding with limited significance.^21^ In this context, the authors align with the viewpoint that regards it as a unique pathological subtype of EPC. This may represent a pattern of malignant transformation in EP, but the exact mechanisms behind it require further research and investigation. Similar to squamous differentiation, the authors also observed statistically significant differences in spindle cell change. In contrast to the 4% reported in the previous study, the authors observed spindle cell change in 18.2% of these cases.^5^ Robson et al. considered this type of EPC as a spindle cell variant of porocarcinoma and proposed a spectrum that may exist between a pure spindle cell porocarcinoma and tumors with more classical morphology.^5^ This type of EPC can express a high degree of heterogeneity and malignancy.^22^

The authors also suspect that tumors that differentiate into apocrine glands with more complex components, including hair, are more susceptible to malignant transformation. The authors have therefore observed decapitate secretion and sebaceous differentiation in these cases. However, we have not found significant differences in these cases to support this hypothesis. Delicate fibrovascular stroma and sharp demarcation between normal keratinocytes and poroma cells are important pathological features of poroma and were also common in the EPC group in this study, with no significant difference between the two groups.

In conclusion, the present study highlights the differences in clinicopathological features between EP and EPC patients. EPC occurs more frequently in older patients, and exposed sites, and is more likely to be ulcerated and to be larger. Histologically, the authors found infiltrative growth patterns, asymmetric tumor architecture, ulceration, central necrosis, squamous differentiation, spindle cell changes, and diffuse inflammatory infiltration significantly more frequent in the EPC group. The findings of this study may help to identify potential factors associated with the malignant transformation of EP and assist clinicians in diagnosis and management.

## Financial support

None declared.

## Authors’ contributions

Qin-Xiao Wang: Conceptualization; data curation; methodology; formal analysis; visualization; validation; writing-original draft.

Si-Yu Luo: Data Curation; investigation; visualization.

Kai-Yi Zhou: Data Curation; project administration; validation.

Xiao Shen: Resources; Supervision; writing-review & editing.

Sheng Fang: Conceptualization; Methodology; resources; supervision; writing-review & editing.

## Conflicts of interest

None declared.
